# Awareness and Perception of Dental, Medical, and Paramedical Students Toward the Use of Clear Aligners in Orthodontic Treatment in Belagavi, India

**DOI:** 10.7759/cureus.64737

**Published:** 2024-07-17

**Authors:** Trupti Sadhunavar, Amit Nilgar, Varkey Nadakkavukaran Santhosh, Sagar Jalihal, Siva Shankkari

**Affiliations:** 1 Orthodontics, KLE Vishwanath Katti Institute of Dental Sciences, KLE Academy of Higher Education and Research, Belagavi, IND; 2 Orthodontics and Dentofacial Orthopedics, KLE Vishwanath Katti Institute of Dental Sciences, KLE Academy of Higher Education and Research, Belagavi, IND; 3 Public Health Dentistry, KLE Vishwanath Katti Institute of Dental Sciences, KLE Academy of Higher Education and Research, Belagavi, IND

**Keywords:** health professionals, perception, orthodontic treatment, clear aligners, awareness

## Abstract

Background

The advent of clear aligners represents a significant shift in orthodontic treatment, offering an aesthetic and convenient alternative to traditional braces and helping maintain better oral hygiene as it is removable. This study investigates the awareness and perception of clear aligners among dental, medical, and paramedical students in Belagavi, India.

Methodology

A cross-sectional, observational study was conducted in February 2024 among students from a private medical university in Belagavi. A validated questionnaire (content validity ratio = 0.88; Cronbach’s alpha coefficient = 0.86), developed through expert consultation and pilot testing, assessed the awareness and perception of students on clear aligners. Simple random sampling was used to select 480 participants. Data were analyzed using the chi-square test, analysis of variance, Pearson correlation coefficient, and binary logistic regression.

Results

The study found that awareness and perception of clear aligners were the lowest among medical and paramedical students in comparison with dental students. A positive linear correlation was seen between awareness and perception scores. When specialty was taken into consideration, medical/dental students were 2.55 times more aware and had 2.78 times more positive perceptions toward clear aligners compared to paramedical students.

Conclusions

There was a notable disparity in the awareness and perception of clear aligners among dental, medical, and paramedical students. Medical and paramedical students displayed lower awareness and unfavorable perception toward clear aligners in comparison with dental students.

## Introduction

Orthodontic treatment has witnessed a paradigm shift with the advent of clear aligners, a pioneering alternative to traditional fixed appliances [1]. The conventional production procedure for clear aligners consists of the following four stages: obtaining the initial dental anatomy, adjusting teeth, three-dimensional (3D) printing, and thermoforming. Thermoforming clear aligners involves heating a thermoplastic sheet, molding it over a 3D model of the patient’s teeth, and then trimming and finishing it for precise fit and comfort. However, to bypass the multiple steps involved, direct printing has been introduced. It uses 3D technology to create customized dental devices from digital scans [2]. These virtually invisible aligners, fabricated from transparent thermoplastic materials, offer a discrete and convenient solution for correcting malocclusions and achieving proper dental alignment [1]. Unlike conventional braces, which comprise brackets and wires, clear aligners are a series of custom-manufactured, computer-generated appliances that gradually reposition teeth through a sequence of progressive aligner stages [3]. Patients can maintain their oral hygiene more easily because the aligners are removable [4].

The clinical efficacy of clear aligners is well-established in mild-to-moderate malocclusions, and their increasing popularity can be attributed to several inherent advantages [5,6]. The superior aesthetics of these aligners address the cosmetic concerns of patients seeking a less conspicuous treatment modality [7]. The removable nature of clear aligners allows for uninterrupted oral hygiene practices and dietary habits, minimizing the disruptions often associated with fixed appliances [8,9]. It reduces the risk of plaque accumulation and subsequent periodontal complications. Additionally, the precise fit of clear aligners minimizes gingival impingement, thereby promoting a healthier periodontal environment compared to traditional fixed appliances [10]. Patients also benefit from the convenience of being able to remove the aligners during meals, allowing for normal eating habits without dietary restrictions. Overall, the combination of aesthetic appeal, comfort, hygiene, convenience, and enhanced accessibility makes clear aligners an effective and desirable choice for individuals seeking orthodontic treatment while maintaining a high level of oral health and quality of life.

As the demand for clear aligners continues to surge, it is imperative to assess the awareness and perception of this innovative technology among future healthcare professionals, including medical and paramedical students [11]. These individuals play a pivotal role in promoting oral health and educating patients about various treatment options. As they are often the first point of contact with patients, it is crucial for them to have a comprehensive understanding of clear aligners. By possessing knowledge about this innovative treatment modality, they can play a pivotal role in educating patients and providing valuable insights. Their knowledge and perception of clear aligners can significantly influence the adoption and implementation of this technology within the broader community.

This study aims to elucidate the current level of awareness and perception of clear aligners among dental, medical, and paramedical (physiotherapy and nursing) students in Belagavi. By exploring their awareness of this innovative treatment modality, the research seeks to identify potential gaps or misconceptions that may exist among this demographic. Additionally, the study aims to uncover factors influencing the students’ perceptions, such as affordability, accessibility, and perceived benefits or drawbacks of clear aligners compared to traditional braces.

## Materials and methods

Study design, duration, and setting

This was a cross-sectional, observational study conducted among dental, medical, and paramedical (physiotherapy and nursing) students in a private medical university in Belagavi, India, in accordance with the Strengthening The Reporting Of Observational Studies In Epidemiology (STROBE) reporting guidelines. The study was conducted in February 2024.

Ethical clearance and informed consent

Ethical clearance was obtained from the Institutional Research Ethics Committee of KLE Vishwanath Katti Institute of Dental Sciences (approval number: EC/NEW/INST/2021/2435). The objective of the study was clearly explained and informed consent was obtained from all study participants.

Questionnaire development

The questionnaire development went through a six-stage process beginning with the designing of a conceptual framework based on experts’ opinions and existing literature. The item pool was generated with 10 questions in both awareness and perception construct. This was followed by a focus group discussion with eight subject experts and five representative participants. Continuous evaluation of the questionnaire was done by the experts. This was followed by cognitive interviewing of the representative participants. The addition and removal of specific items were carried out based on their relevance, and a refined questionnaire was prepared.

Pilot testing

A pilot study was conducted among 20 medical students (13 females and 7 males) who were representative of the target population. It was aimed at determining any ambiguity in the wording or difficulty in comprehension of the questions by the students. Two questions in the awareness construct and three questions in the perception construct were found to be unclear for the participants and modification was done based on their feedback to improve the clarity of the questionnaire.

Validity and reliability assessments

The percentage agreement among a panel of five subject experts was obtained to determine the face validity which was found to be 83%. Additionally, the content validity ratio was determined for the questionnaire, and it was found to be a valid tool, with a content validity ratio of 0.88. The reliability of the questionnaire was analyzed using Cronbach’s alpha coefficient, which was estimated to be 0.86, indicating high internal consistency. Considering these findings, the questionnaire was found to be reliable and appropriate for gathering the desired study data.

Sample size estimation and sampling technique

The pilot study found that 70.69% of female and 51.85% of male students were aware of clear aligners. The allocation ratio for females to males was 3:1. Based on the findings of the pilot study, the minimum sample size for the current study was found to be 472 considering type-I (α) error of 0.05 and power (1-β) of 0.95 using G*Power statistical software (Version 3.1.9.4.). The final sample size was rounded off to 480 students.

A simple random sampling technique was employed in this study. The list of medical and paramedical students from the medical university was used as the sampling frame. The students were recruited by the random number table method. The findings from the pilot study were not included in the final analysis.

Questionnaire characteristics

A self-administered questionnaire was distributed to the students containing 20 close-ended questions in English. The first 10 questions aimed to assess the students’ awareness of clear aligners, while the remaining 10 questions evaluated their perception of clear aligners. It also recorded demographic details of the participants. A five-point psychometric Likert scale (scored from 1 to 5) was employed and the summative scores were calculated for every student. Students with a mean score of 40 and above were considered to be both aware and possess a positive perception toward clear aligners.

Data collection

The data collection process was performed by a single investigator. During regular college hours, the investigator visited the classrooms of the students and distributed physical copies of the questionnaire. The students were allotted 15 minutes to complete the questionnaire under the supervision of the investigator and two volunteers. This supervised approach ensured the integrity and reliability of the collected data, preventing any potential compromises.

Statistical analysis

Data entry was carried out in Microsoft Excel 2019 and analyzed using SPSS® Statistics Version 21 (IBM Corp., Armonk, NY, USA). Descriptive statistics were computed to find the mean, standard deviation, frequency, and percentages. The normality of the data was analyzed using the Kolmogorov-Smirnov test and the distribution was found to be normal. The chi-square test was employed to determine the differences in the responses to awareness and perception questions. The awareness and perception scores were compared using the analysis of variance test to find any significant differences among the various groups. The Pearson correlation coefficient test was performed to find the correlation between awareness and perception of participants. Binary logistic regression analysis was also carried out. All the above-mentioned tests were conducted at a 95% confidence interval and a 5% significance level.

## Results

**Figure 1:**
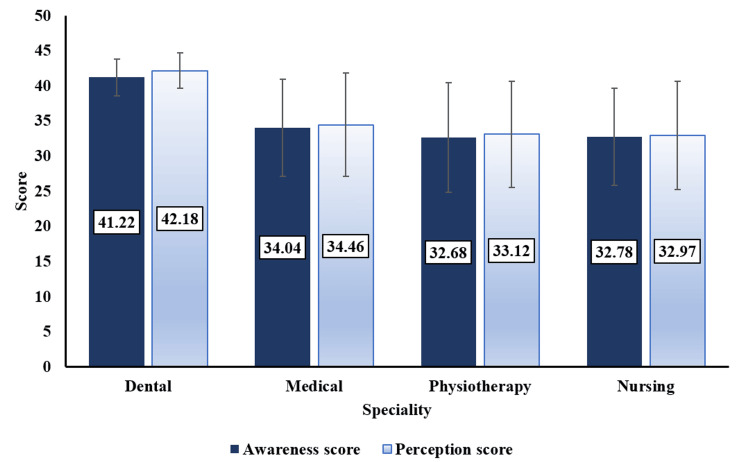
Comparison of awareness and perception score among various specialties.

The present study included a total of 480 students, with 120 students belonging to each of the four specialties studied. Table 1 shows the demographic profile of the study participants. The mean age of the students included in the study was 21.31 ± 3.06 years, and the majority (71.8%) were females. The frequency distribution of the responses to both awareness and perception-based questions are presented in Table 2 and Table 3. The majority (76.2%) of students were aware of clear aligners as an option for orthodontic treatment while only a small proportion (16.6%) were not aware. It was observed that a large proportion of students (65.3%) were aware of various brands of clear aligners in the market while 17.5% were not aware. Among the students, 67.9% agreed that clear aligners are affordable and accessible while 19.2% found them not affordable. Moreover, 67.7% of the students agreed patients choose clear aligners over traditional braces because of aesthetics and comfort, while 16.0% of the participants disagreed. Table 4 and Figure 1 present the mean awareness and perception scores of study participants on clear aligners. The highest awareness (41.22 ± 2.62) and perception (42.18 ± 2.51) scores were found among dental students while the lowest awareness and perception scores were found among physiotherapy (32.68 ± 7.79) and nursing (32.97 ± 7.70) students. A statistically significant difference was found in both the awareness and perception scores among the participants in various specialties (p ≤ 0.001).

**Table 1:** Demographic profile of the participants. Age is expressed as mean ± SD. Gender is expressed as frequency and percentage (in parentheses). SD = standard deviation

Demographic profile	Dental, n (%)	Medical, n (%)	Physiotherapy, n (%)	Nursing, n (%)
Gender				
Male	39 (32.5%)	48 (40%)	35 (29.2%)	13 (10.8%)
Female	81 (67.5%)	72 (60%)	85 (70.8%)	107 (89.2%)
Total	120 (100.0%)	120 (100.0%)	120 (100.0%)	120 (100.0%)
Age (mean ± SD)	22.13 ± 2.87	24.13 ± 3.33	19.92 ± 1.38	19.04 ± 0.82

**Table 2:** Frequency distribution of responses to awareness-based questions on clear aligners among participants. All values are expressed as frequency and percentage (in parentheses). Statistical test used: chi-square test. Level of significance: p-values ≤0.05 are considered statistically significant.

Awareness questions	Very unaware	Unaware	Neither aware nor unaware	Aware	Very aware	P-value
How would you rate your awareness of clear aligners as an option for orthodontic treatment?	17 (3.5%)	63 (13.1%)	34 (7.1%)	349 (72.7%)	17 (3.5%)	<0.001*
How familiar are you with the various brands or manufacturers of clear aligners available in the market?	13 (2.7%)	71 (14.8%)	83 (17.3%)	271 (56.5%)	42 (8.8%)	<0.001*
How well-informed do you feel about the working mechanism of clear aligners compared to traditional braces?	17 (3.6%)	67 (14.0%)	60 (12.6%)	307 (64.2%)	27 (5.6%)	<0.001*
How knowledgeable are you about the advantages of using clear aligners over traditional braces?	25 (5.2%)	57 (11.9%)	90 (18.8%)	262 (54.7%)	45 (9.4%)	<0.001*
How informed are you about the specific dental conditions for which clear aligners are particularly effective?	22 (4.6%)	96 (20.0%)	53 (11.0%)	238 (49.6%)	71 (14.8%)	<0.001*
How confident are you in your knowledge of whether clear aligners can be used for both minor and complex orthodontic cases?	23 (4.8%)	55 (11.5%)	88 (18.3%)	260 (54.2%)	54 (11.3%)	<0.001*
To what extent do you believe you understand the typical steps involved in the process of obtaining and using clear aligners for orthodontic treatment?	18 (3.8%)	72 (15.0%)	82 (17.1%)	221 (46.0%)	87 (18.1%)	<0.001*
How aware are you of any limitations or contraindications associated with the use of clear aligners?	23 (4.8%)	91 (19.0%)	83 (17.3%)	242 (50.4%)	41 (8.5%)	<0.001*
How well-versed are you in the factors to consider when determining the suitability of clear aligners for a patient’s orthodontic treatment plan?	17 (3.5%)	87 (18.1%)	73 (15.2%)	269 (56.0%)	34 (7.1%)	<0.001*
To what extent are you aware of clear aligners being considered a viable alternative to traditional braces in orthodontic treatment?	22 (4.6%)	58 (12.1%)	82 (17.1%)	285 (59.4%)	33 (6.9%)	<0.001*

**Table 3:** Frequency distribution of responses to perception-based questions on clear aligners among participants. All values are expressed as frequency and percentage (in parentheses). Statistical test used: chi-square test. Level of significance: p-values ≤0.05 are considered statistically significant.

Perception questions	Strongly disagree	Disagree	Neither agree or disagree	Agree	Strongly agree	P-value
How strongly do you perceive clear aligners as a viable option for orthodontic treatment?	34 (7.1%)	44 (9.2%)	71 (14.8%)	303 (63.1%)	28 (5.8%)	<0.001*
To what extent do you agree or disagree that clear aligners are widely accepted by orthodontic patients compared to traditional braces?	14 (2.9%)	66 (13.8%)	77 (16.0%)	290 (60.4%)	33 (6.9%)	<0.001*
How much do you agree or disagree that patients choose clear aligners over traditional braces for reasons such as aesthetics and comfort?	26 (5.4%)	51 (10.6%)	78 (16.3%)	273 (56.9%)	52 (10.8%)	<0.001*
Do you feel that patients often have misconceptions or concerns about clear aligners that impact their decision-making process?	21 (4.4%)	64 (13.3%)	69 (14.4%)	288 (60.0%)	38 (7.9%)	<0.001*
How strongly do you agree or disagree that clear aligners are as effective as traditional braces in achieving desired orthodontic outcomes?	28 (5.8%)	64 (13.3%)	69 (14.4%)	260 (54.2%)	59 (12.3%)	<0.001*
Do you feel there is a need to increase the marketing for clear aligners?	13 (2.7%)	76 (15.8%)	74 (15.4%)	245 (51.0%)	72 (15.0%)	<0.001*
Do you feel clear aligners are accessible and affordable for patients?	31 (6.5%)	61 (12.7%)	62 (12.9%)	254 (52.9%)	72 (15.0%)	<0.001*
How strongly do you agree or disagree that patients who undergo orthodontic treatment with clear aligners are generally satisfied with the outcomes?	12 (2.5%)	75 (15.6%)	70 (14.6%)	261 (54.4%)	62 (12.9%)	<0.001*
To what extent do you agree or disagree that dental professionals in the Belagavi district possess the necessary training and expertise to use clear aligners effectively?	17 (3.5%)	50 (10.4%)	74 (15.4%)	288 (60.0%)	51 (10.6%)	<0.001*
Do you feel that there is room for improvement in clear aligner technology to enhance orthodontic treatment outcomes?	11 (2.3%)	66 (13.8%)	83 (17.3%)	278 (57.9%)	42 (8.8%)	<0.001*

**Table 4:** Comparison of awareness and perception of clear aligners among various specialties. All values are expressed as mean ± SD. Statistical test used: one-way analysis of variance followed by Tukey’s post-hoc test. Level of significance: p-values ≤0.05 are considered statistically significant. Groups with the Greek letter α in superscript show statistical significance with dental. SD = standard deviation

Specialty	Dental (n = 120)	Medical (n = 120)	Physiotherapy (n = 120)	Nursing (n = 120)	P-value
Awareness score (mean ± SD)	41.22 ± 2.62	34.04 ± 6.88^α^	32.68 ± 7.79^α^	32.78 ± 6.92^α^	<0.001*
Perception score (mean ± SD)	42.18 ± 2.51	34.46 ± 7.35^α^	33.12 ± 7.52^α^	32.97 ± 7.70^α^	<0.001*

**Figure 2:**
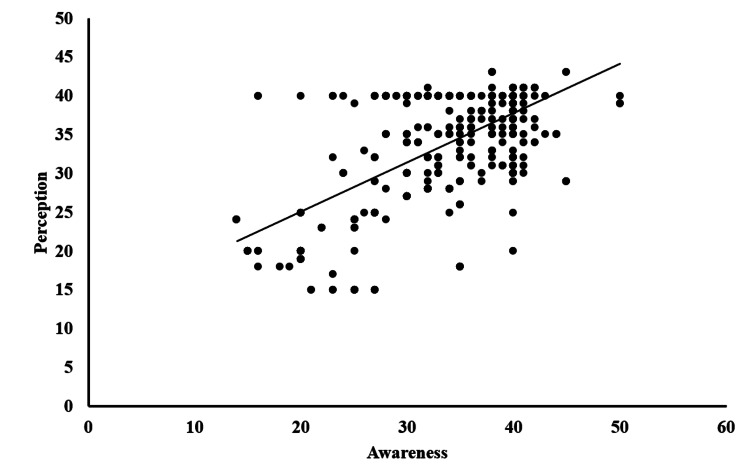
Scatter plot showing the correlation between awareness and perception of clear aligners among students.

There was a positive linear correlation between awareness and perception scores (r = +0.706; p ≤ 0.001). Figure 2 presents the scatter plot illustrating the correlation between awareness and perception. Binary logistic regression analysis revealed that males were 1.38 times more aware and had 1.06 times more positive perceptions toward clear aligners when compared to females. When specialty was taken into consideration, medical/dental students were 2.55 times more aware and had 2.78 times more positive perceptions toward clear aligners compared to paramedical students (Table 5).

**Table 5:** Association between awareness and perception with demographic characteristics of study participants. Paramedical specialty includes nursing/physiotherapy. Reference category in gender: female; in specialty: paramedical. Statistical test used: binomial logistic regression. Level of significance: p-values ≤0.05 are considered statistically significant. OR = odds ratio; SE = standard error; CI = confidence interval

Parameters	Awareness of clear aligners				Perception of clear aligners			
	OR	SE	95% CI	P-value	OR	SE	95% CI	P-value
Gender								
Female	Reference	-	-	0.348	Reference	-	-	0.854
Male	1.382	0.345	0.703–2.719		1.059	0.312	0.574–1.953	
Specialty								
Paramedical	Ref	-	-	0.003*	Ref	-	-	<0.001*
Dental/Medical	2.552	0.315	1.378–4.727		2.783	0.304	1.535–5.045	

## Discussion

Clear aligners have gained widespread acceptance and preference among patients for their inconspicuous and visually appealing design, enabling individuals to straighten their teeth without the conspicuous metal components of conventional braces [12]. They are typically made from a biocompatible material, such as polyurethane resin or polyethylene terephthalate glycol. These materials are chosen for their durability, flexibility, and aesthetic properties [13,14]. Several studies have been conducted on biodegradable aligner materials [15]. Hence, it is necessary for medical and paramedical students to be aware of this new treatment modality, as future healthcare professionals who may encounter patients seeking orthodontic treatment. Their knowledge of clear aligners can help guide patients toward the most suitable and preferred options such as clear aligners [16]. The present study aimed to assess the awareness and perception of medical and paramedical students toward clear aligners as an orthodontic treatment modality.

In the present study, there was a relatively low level of awareness about clear aligners among medical and paramedical students. In contrast, dental students exhibited a significantly higher level of awareness regarding the same. These findings are consistent with the study conducted by Shivlani et al., which reported low awareness about clear aligners among graduates in fields such as Ayurveda, physiotherapy, and nursing [11]. However, our findings are in contrast to those of Pawar et al., where the majority of participants demonstrated prior knowledge about clear aligners [17]. This discrepancy may be attributed to differences in the demographics of the study populations, or variations in the curricula of the respective educational programs. The varying levels of awareness highlighted by these studies underscore the need for more uniform and comprehensive educational strategies to ensure that all healthcare students, regardless of their specialty, are well-informed about the latest advancements in orthodontic treatments such as clear aligners.

When overall awareness of the students was considered, a majority of them were aware of the various brands that are available in the market. They were also aware of the mechanism behind aligner technology and the advantages of using clear aligners over conventional treatment modalities. One potential reason why many were aware of clear aligners could be the increasing popularity and marketing of clear aligner treatments such as Invisalign [18]. With the widespread availability of information on the internet, social media, and through word-of-mouth, it is possible that many students in the study had been exposed to the marketing and information about clear aligners, leading to an increased awareness of their advantages over conventional treatment modalities.

When considering student perceptions, a similar trend emerged. Students from dental specialties demonstrated a positive perception of clear aligners, whereas students from medical and paramedical specialties did not share this favorable view. These findings aligned with that of Shivlani et al. and Linjawi et al. [11,19]. The poor perception among the latter groups may be attributed to the strong correlation observed between awareness and perception. This suggests that the limited awareness of clear aligners within the medical and paramedical cohorts likely contributes to their less favorable perceptions. Enhancing educational initiatives and informational campaigns specifically targeting these groups could bridge this awareness gap. By increasing their understanding of the efficacy, benefits, and technological advancements associated with clear aligners, it is plausible that these students would develop a more positive perception, akin to their counterparts in dental specialties. This highlights the critical role of targeted awareness programs in shaping informed perceptions across different fields of study.

When the overall perception of the students was considered, a majority viewed clear aligners as an affordable treatment option, contrary to the common perception of them being expensive. This positive perception could facilitate broader adoption and access to clear aligner therapy, especially in regions where finances are less of a barrier for some patients. Additionally, the study revealed that most students believed patients prefer clear aligners over traditional braces due to their superior aesthetics and comfort. This finding aligns with existing literature, which highlights the aesthetic appeal and convenience of clear aligners as significant factors driving their growing popularity among patients seeking orthodontic treatment [17].

The study revealed that a significant majority of the students perceive a considerable need for enhanced marketing efforts aimed at promoting clear aligners as a viable option for orthodontic treatments. This sentiment underscores a potential gap in awareness and understanding of the benefits and availability of clear aligners compared to traditional orthodontic methods [20]. The current marketing strategies may not be sufficiently reaching or engaging the target demographic, indicating an opportunity for more comprehensive and targeted marketing campaigns [21]. Such initiatives could focus on educating potential patients about the efficacy, convenience, and aesthetic advantages of clear aligners, thereby potentially increasing their adoption, and improving overall patient outcomes in orthodontic care [22]. The majority of students expressed the opinion that there is considerable room for improvement in clear aligner technology to enhance orthodontic treatment outcomes. Despite acknowledging these benefits, students still reported a lack of confidence in current aligner technology, indicating that existing limitations may impact their overall perception and trust in these orthodontic solutions [23].

The results of the study demonstrated that medical and paramedical students exhibited significantly lower awareness and less positive perceptions toward clear aligners compared to their dental counterparts. This disparity could also be attributable to the differences in curriculum and exposure to orthodontic concepts among these disciplines. Nonetheless, it underscores the need for enhanced education and training initiatives to bridge this gap and ensure that all future healthcare professionals possess adequate knowledge and understanding of this innovative treatment modality.

Limitations

The present study has certain limitations. The cross-sectional nature of the study precludes the establishment of causal relationships between the variables investigated. Furthermore, the study was conducted in a single medical university, which may limit the generalizability of the findings to other geographical regions or educational institutions with different curricula or student demographics. Another potential limitation is the self-reported nature of the data, which could be subject to response biases or social desirability biases.

Future recommendations

Incorporating comprehensive educational modules on clear aligners and other contemporary orthodontic treatment modalities into the curricula of medical and paramedical programs would ensure that future healthcare professionals are well-informed about the latest advancements and can provide accurate information to their patients. It is crucial to encourage interdisciplinary collaborations and knowledge-sharing initiatives between dental and medical/paramedical disciplines. This could involve joint seminars, workshops, or case-based learning sessions, fostering a better understanding of clear aligners and their applications across various healthcare domains. Targeted awareness campaigns and educational resources tailored specifically for medical and paramedical students should be developed. These campaigns could leverage digital platforms, social media, and interactive learning tools to effectively disseminate information about clear aligners and address any misconceptions or knowledge gaps. Further research is needed to explore the factors influencing the perceptions and attitudes of medical and paramedical students toward clear aligners. Qualitative studies or mixed-methods approaches could provide deeper insights into the underlying beliefs, concerns, or barriers that shape their perceptions, enabling the development of targeted interventions or educational strategies. It is also important to encourage the involvement of medical and paramedical students in clinical observerships or rotations within orthodontic settings. Hands-on exposure and direct observation of clear aligner treatment cases could enhance their practical understanding and appreciation of this treatment modality. By implementing these recommendations, educational institutions and healthcare organizations can contribute to enhancing the knowledge and positive perceptions of clear aligners among future healthcare professionals, ultimately benefiting patient care and facilitating informed decision-making regarding orthodontic treatment options.

## Conclusions

The study revealed a relatively low awareness of clear aligners among medical and paramedical students, while dental students displayed the highest awareness and had the most positive perceptions. While most students recognized the affordability, accessibility, and aesthetic benefits of clear aligners, efforts are needed to further increase awareness and improve perceptions, especially among paramedical students. Enhancing education on clear aligners across healthcare disciplines, promoting interdisciplinary collaboration, conducting awareness campaigns, providing hands-on exposure, and continued research can foster a well-informed workforce to drive broader acceptance and accessibility of this innovative orthodontic treatment modality.
